# Selective and optimal dynamic pricing strategy for residential electricity consumers based on genetic algorithms

**DOI:** 10.1016/j.heliyon.2022.e11696

**Published:** 2022-11-16

**Authors:** Salma Taik, Bálint Kiss

**Affiliations:** Department of Control Engineering and Information Technology, Budapest University of Technology and Economics, 1111, Budapest, Hungary

**Keywords:** Consumer behavior model, Consumers categorization, Consumption data clustering, Demand-side management program, Parameter uncertainty, Price sensitivity, Time-of-Use electricity tariffs

## Abstract

The continuous increase in residential population implies an increase in electricity demand. The demand prediction performed by the utility company (UC) to schedule and plan the next energy purchase from the market may not be efficient since the actual demand consistently exceeds the supply. To use the energy production infrastructure efficiently and decrease the grid system's overload, the UC seeks to match the demand with the supply. Dynamic pricing is a simple yet effective way to influence consumption as price-sensitive consumers may reschedule the operation of some of the appliances. This paper presents a novel, consumption data-driven, selective and dynamic Time-of-Use (ToU) electricity pricing strategy applicable by the UC for residential electricity consumers. The method consists of clustering real consumption data using the k-means clustering technique and heuristically categorizing clusters based only on their consumption data to simplify the design of the ToU tariffs and limit the number of different tariffs in the same population. The method includes a heuristic determination of the time period for the ToU tariff changes for each category. The ToU tariff parameters are determined by minimizing a single cost function using genetic algorithms and considering all consumer clusters such that the consumer behavior model is based on price elasticity. The optimal ToU tariffs result in decreasing the demand in power peaks by targeting the overconsumer categories in the latter. Implementing the ToU tariff results in a profit margin distributed among the UC and the consumer. Moreover, the optimal prices guarantee positive gains for both of them with the presence of a wide range of parameter uncertainties. The proposed pricing strategy necessitates the presence of consumption data only, which implies the conservation of the consumers' privacy. Real consumption data obtained for two towns in Hungary over three years is used to show the efficiency of the proposed pricing strategy.

## 1 Introduction

Although the household electricity demand in developed countries and regions such as the USA and the EU has remained relatively constant recently, the electrification of transport due to the growing number of electric vehicles (EV) will increase electricity peak demands [1]. The augmentation of EVs' number connected to the power grid may result in line congestion and voltage limit violations [2]. The latter can also be caused due to the high penetration of distributed renewable electricity generators that should be appropriately regulated to maintain the stable operation of the grid. Meanwhile, a steady increase in household electricity demand can also be observed in developing countries, and the growth of population centers contributes to the concentration of electric consumption in a geographically limited part of the infrastructure.

As the power production and distribution industries undergo transformation due to disruptive new technologies and business models, each utility company (UC) still faces the challenge of supplying electricity reliably and cost-effectively to the consumers with limited impact on the environment. The increase of the production capacity and the distribution infrastructure to meet higher peak demands is expensive, time-consuming, and results in undesired environmental impact [3]. Improved prediction and dynamic control of the time-distribution of energy consumption is considered a better option, and it can also accommodate the use of clean energy resources. An important aim of controlling the consumption is to continuously and accurately match the prediction-based production with limited deviation as the instantaneous production of extra energy is expensive [4]. Consumption prediction is usually based on previously recorded consumption patterns.

As smart grids allow real-time consumption monitoring, many UCs already propose demand-side response (DSR) programs to their clients to influence their consumption patterns providing benefits both to the UC and the consumers [5]. There are two major DSR programs: incentive-based programs and price-based programs [6]. This paper investigates a price-based DSR strategy for consumers in residential areas. In response, the consumer can save on his monthly bills by rearranging his consumption profile (i.e., shifting loads from peak hours) with the help of an automated algorithm [4]. In our setup, the prices are optimized using simple and identifiable consumer behavior models (CBMs) for multiple consumer categories. The DSR program allows the UC to minimize the additional energy purchases with a spot market price and reduce costs.

Various dynamic pricing schemes have been introduced in the literature; the reader may refer to the papers [7] or [8] for a comprehensive survey. Methods scrutinized in the literature include real-time pricing [9], [10], critical peak pricing [11], [12], and time-of-use (ToU) tariffs [13], [14]. Real-world experiments with residential and industrial consumers are also reported in [15]. ToU tariffs are commonly adopted in DSR programs since the prices change less frequently compared to the other dynamic pricing strategies, and consumers prefer it because they easily understand its structure and they are willing to pay [16]. ToU tariffs are often characterized by two major time segments [14]; on-peak and off-peak. Electricity prices are higher on on-peak and lower on off-peak.

The optimal design of ToU tariffs has also been intensively studied recently. Authors of [17] surveyed optimal ToU tariff structures. Rahman et al. [18] proposed four different tariff schemes for the residential sector; however, it is suitable for low-income consumers only. Dong et al. [19] report a robust benefit of ToU tariffs over the flat-rate tariff. In most reported cases, the ToU prices are optimized, but periods are defined empirically and generally applied the same way to all consumers. Jang et al. [16] presented a similar clustering and categorization methods that has the same purposes. However, their method requires six different features that the consumer has to provide, such as their income, the status of their family members, etc., that were collected thanks to a one-to-one individual survey. Samadi et al. [20] propose a pricing algorithm to reduce the peak-to-average ratio of the aggregated load demand if the UC is uncertain about consumer responsiveness. The electricity price model presented in [21] calculates flat and dynamic prices where the extra energy generation and purchase costs are divided equally as an award among the consumers. Monfared et al. [22] present a dynamic pricing framework based on the electricity value for consumers. However, due to its formulation, the model sets hard limitations on the benefits of the consumers and the UC.

The following points summarize the limitations of the existing literature. 1) The DSR program must guarantee that the UC and the consumers robustly benefit from an improved overall consumption pattern even if the consumers' reaction to the tariffs is subject to uncertainty. 2) Consumers with different consumption characteristics usually coexist in an area supplied by the same UC. Hence, consumer classification and analysis of their ability to reschedule consumption are necessary. 3) Categorizing consumers based on their load requires additional private information that may violate the consumers' privacy; hence, they might deny participating in a DSR strategy. 4) Each consumer class contributes differently to the overall power peak that the UC seeks to smoothen. This implies that applying different tariffs (i.e., different prices with varying segments of time) to each consumer class may result in a better optimum for the UC and the entire consumer community. 5) The challenge is to determine the shape of the optimal ToU tariff within the optimization to capture the fluctuation of the demand curve of each consumer class. Hence, adopting fixed time blocks cannot be applied to all classes. 6) Predefining constraints on possible minimum and maximum ToU tariffs in each period may limit the calculation range, and the optimal ToU tariffs may not be reached.

**Figure 1:**
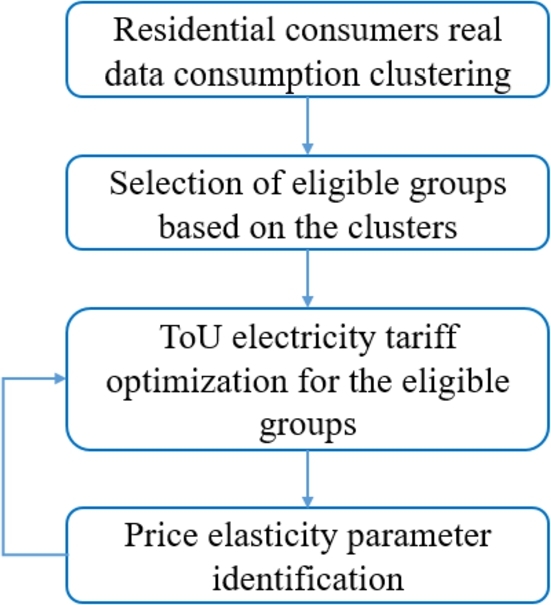
Steps of the proposed framework.

This paper presents a selective, cluster-based ToU tariff strategy that robustly guarantees all stakeholders' profits using a genetic algorithm-based optimization and considering a simple, price-sensibility model with identifiable parameters. Fig. 1 shows the steps of the proposed methodology.

The behavior of the consumers is modeled with price elasticity parameter sets which are different for each consumer category and can be identified based on consumption data. Although a different optimal ToU tariff is obtained for each category, a single objective function ensures that the DSR program benefits are distributed between the UC and the consumers. For that purpose, weighting factors are introduced in the objective function, which is a novel approach focusing on all stakeholders' benefits to the best of our knowledge. The optimization problem is carried out using genetic algorithms (GA) [23] due to the non-linearity nature of the problem.

The contributions of this paper are summarized and listed as follows:

1) The proposed approach systematically established consumer categories based exclusively on consumption data without collecting extra consumer-related specific information (e.g., income, appliances in the household, location, etc.); hence, their privacy is not deprived.

2) It involves designing different ToU tariffs in one population on top of the consumer classification and eligible classes selection giving more flexibility to the benefits of the consumers and the UC. The eligible categories for the ToU tariff implementation are those able to reschedule a part of their demand in different periods. Hence, a study is presented to validate the categories that can participate in the demand-side management program.

3) The optimal ToU tariff structure design considers the behavior of the consumer population and should target the categories that contribute the most to the overall power peak.

4) The ToU tariff blocks are determined dynamically for each consumer category and labeled (i.e., off-peak, mid-peak, and on-peak) according to a heuristic algorithm that considers the average consumption of each category.

The proposed models result in an improved optimal consumption pattern in which its profits are balanced optimally on the UC and the consumers. The overall power peak is reduced by successfully targeting its high contributor. The proposed pricing strategy's profitability robustness is shown against the parameter uncertainty of the consumer behavior model. It can be seen that the profitability is still robust even when the ToU tariff is not optimal. However, the optimal ToU tariff ensures a positive profit for the UC and the consumers in a significant range of uncertainties. Our approach is illustrated using real consumption data collected in two towns in Hungary for three years. The implementation of the proposed pricing strategy will ensure a reduced overall power peak which is essential for the sustainability of the grid system. Additionally, the UC will decrease its costs by the reduction of overconsumption and will plan the energy purchase efficiently, which will result in reducing the energy production impact on the environment by scheduling more renewable energy resources operations. On the other hand, the consumer will have an economic incentive to minimize their costs in response to the optimal ToU tariffs.

The rest of the paper is organized as follows. Section 2 presents the consumption data, the clustering technique used in the sequel, and the CBM. Section 3 describes the optimal ToU tariff design, and Section 4 presents the resulting optimal ToU tariffs for the real consumption data. We study the robustness of the optimal ToU tariff in Section 5 and present the identification method to obtain the parameters of the CBM. Section 6 concludes the paper.

## 2 Consumption analysis and modeling

This section presents the analysis of consumption data, the consumer classification and categorization, and the CBM. The dataset used in this paper was provided by one of the utility companies in Hungary and is available from 2016 to 2018. The two cities under study are different in population size. We will refer to the smaller town (around 100,000 inhabitants) as City A and the larger city (approximately 150,000 inhabitants) as City B.

Due to the underlying smart metering infrastructure, the original sampling interval of the electricity consumption is 15 minutes. An hourly aggregation is carried out first to reduce the size of the dataset. Outliers are managed so that consumers with constantly zero (or close-to-zero) consumption are eliminated. Moreover, using interval-based consistency checks, wrong hourly consumption readings are corrected by the previous and next sample average. Next, a weekly averaging of the consumption data of all consumers is performed. Due to seasonal effects, this averaging is executed separately for the period from April until September (referred to as the summer period) and the remaining period as winter. Let $c_{h,d}^{j}$ denote the average consumption of consumer *j* during the hour *h* of the weekday *d* (h=1…24, d=1…7). The normalization of the hourly consumption data is performed to make the electricity load profiles comparable, as the main objective of the ToU tariff is to reduce local consumption peaks, not the overall consumption. The normalized hourly consumption data is denoted by ${\overset{¯}{c}}_{h,d}^{j}=\frac{c_{h,d}^{j}}{\sum_{i=1}^{24}c_{i,d}^{j}}$ $\left({\overset{¯}{c}}_{h,d}^{j}\in [0,1]\right)$. Hence the consumption of each consumer is described by 168 non-negative real numbers for the summer and winter periods.

### 2.1 Clustering and categorization

The clustering of the consumers is performed separately for the winter and the summer periods. The simple and widely used k-means clustering technique is implemented [24], [25]. Considering the summer (respectively winter) period, the consumer *j* is described by the data object $x_{j}=\text{vect}\left\{c_{h,d}^{j}:h=1\ldots 24,d=1\ldots 7\right\}$, where the vect operator creates a unique vector from the elements in the set. The algorithm calculates the distances between a cluster member and the cluster's centroid to assign exclusively each $x_{j}$ to a specific cluster. Various distance measures can be used (Euclidean distance, Minkowski distance, Mahalanobis distance, etc.). We use the squared Euclidean distance $d(x_{j},x_{i})=\sum_{l=1}^{168}{\left(x_{j,l}-x_{i,l}\right)}^{2}$. The clustering algorithm minimizes $J=\sum_{k=1}^{K}\sum_{x_{i}\in Cl_{k}}d(x_{i},c_{k})$, where *K* is the number of the clusters, $Cl_{i}$ denotes the cluster *i* (i=1…K) and $c_{k}$ is the (normalized) centroid of the cluster $Cl_{k}$. By construction, $Cl_{i}\cap Cl_{j}=\emptyset$ (i≠j).

**Figure 2:**
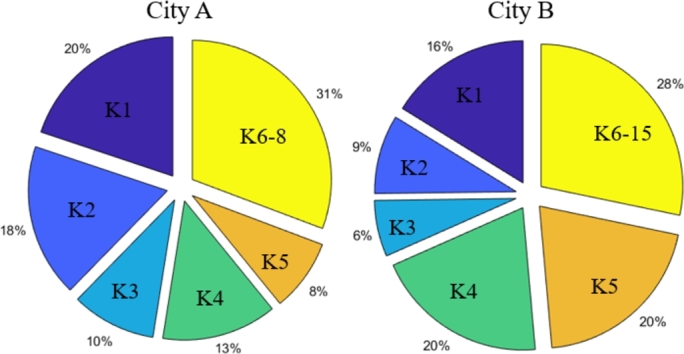
Clusters' share in the total consumption (winter season; left: City A; right: City B).

The number of clusters *K* should be specified a priori to the unsupervised k-means clustering algorithm. If *K* is high, some clusters may contain only a few consumers. Considering the winter season, the number of clusters for the larger city (City B) is set to K=15 whereas K=8 for City A. Fig. 2 shows the energy consumption distribution among the clusters in both cities.

**Figure 3:**
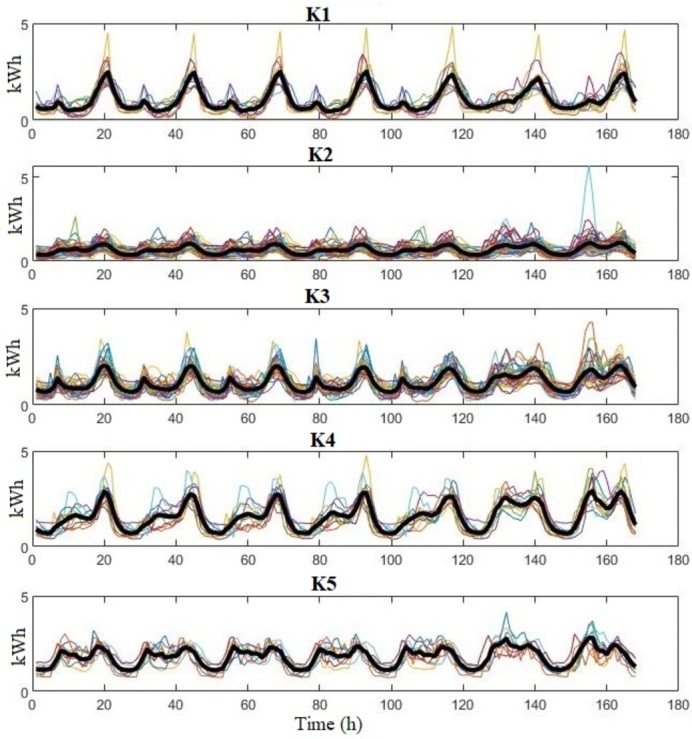
City A: weekly load profiles of clusters K1-K5 (winter season); solid black line: average.

Fig. 3 shows the weekly consumption profiles for clusters K1-K5 (winter season) for City A. Let us remark that the load curves in the figures show the real consumption, although the clustering used normalized values.

The clustering regroups consumers with similar consumption profiles. It is followed by a categorization that allows selecting clusters with consumers eligible for the DSR program and applying different ToU tariffs to consumers with different consumption features. The categorization in existing literature requires different consumer features that should be collected, and it can be private to the consumers. In our pricing strategy, only daily consumption is needed. Consumers with low and constant daily consumption are unlikely to reschedule their energy needs; therefore, they are excluded from the DSR program. The number of categories is lower than the number of clusters to limit the number of different ToU tariffs and avoid applying tariffs to only a few consumers.

Five categories are defined according to the compatibility level with the DSR program: high (1), medium-high (2), average (3), medium-low (4), and low (5). For a larger (resp. smaller) or more (resp. less) diversified consumer population, the number of categories may be increased (resp. decreased). Each cluster belongs to a single category. Next, we specify the categorization algorithm.

The total average daily consumption of each cluster is calculated (denoted by ${\overset{¯}{c}}_{k}$ for the *k*^th^ cluster) with an estimated standard deviation for the consumers in the cluster (denoted by ${\overset{¯}{c}}_{k}^{\sigma }$). The interval $\left[\min_{k}\{{\overset{¯}{c}}_{k}-{\overset{¯}{c}}_{k}^{\sigma }\},\max_{k}\{{\overset{¯}{c}}_{k}+{\overset{¯}{c}}_{k}^{\sigma }\}\right]$ is divided into five equal sub-intervals so that the one with the smallest values is associated with the category 5.

For each cluster, the value ${\overset{¯}{c}}_{k}$ selects an interval and hence a candidate category which is than modified based on ${\overset{¯}{c}}_{k}^{\sigma }$ as specified by Table 1, 5 is being the maximal number designating a category. These modifications express that rescheduling is less likely for clusters where the consumption during the days is small and steady. Let us remark that the categorization process presented here is a simple heuristic, and further considerations may be taken into account.

**Table 1:** Categorization Change of Clusters Based on ${\overset{¯}{c}}_{k}^{\sigma }$.

*Condition*	*Category change*
$0.25<{\overset{¯}{c}}_{k}^{\sigma }$	0
$0.10<{\overset{¯}{c}}_{k}^{\sigma }\leq 0.25$	+1
${\overset{¯}{c}}_{k}^{\sigma }\leq 0.10$	+2

As the consumption of home appliances can usually be rescheduled, the basis of selection is the number of consumption blocks of such appliances that can fit under the average consumption profiles of the categories (e.g., the washing machine's average consumption is 0.5 kWh for two hours, 0.7 kWh for a dishwasher, etc.; for more details, the reader may refer to [26])

**Figure 4:**
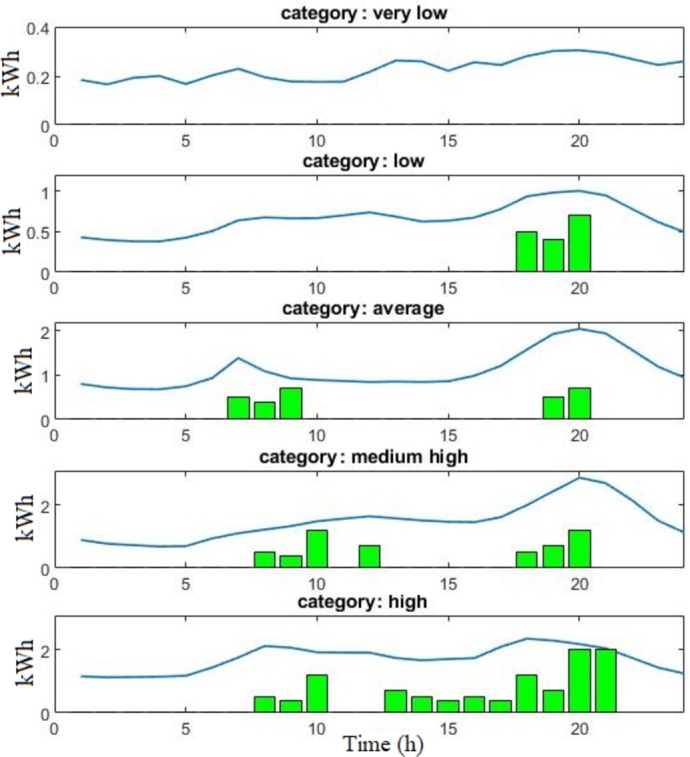
Approximation of the operation of shiftable appliances (green bars) to the average consumption of each category (blue) in one weekday in City A.

Fig. 4 shows each category's average Monday consumption profile (City A, winter season) with the potential operations of the shiftable appliances. The goal is to understand which category accommodates the shiftable appliances. We started by fitting the operation of shiftable appliances under the average consumption curve following realistic possible scenarios. Except for category 5 (very low DSR compatibility), all other consumers can accommodate the operation of the shiftable appliance; hence, they are likely to reschedule the operation of some appliances. Finally, only categories 1, 2, and 3 are selected as the load profile of category 4 (medium-low DSR compatibility) can accommodate only a marginal amount of energy consumption for potential rescheduling. In cities A and B, categories 1, 2, and 3 consume 46% and 34% from the total energy consumed in winter, respectively, and 40% and 36% in summer, respectively.

### 2.2 Consumer behavior model

The responsiveness to changing electricity tariffs is essential to evaluate the resulting change in electricity consumption. Thus, a model to capture the behavior of the consumers is necessary. Earlier studies have already described the consumers' behavior in response to ToU tariff [27], [28]. Consumer psychology considerations [29] show that the consumers' electricity demand decreases nonlinearly when the electricity tariff increases. We adopt a simple and static CBM representing the changes in the consumer's demand with respect to the evolution of the ToU electricity tariff.

**Figure 5:**
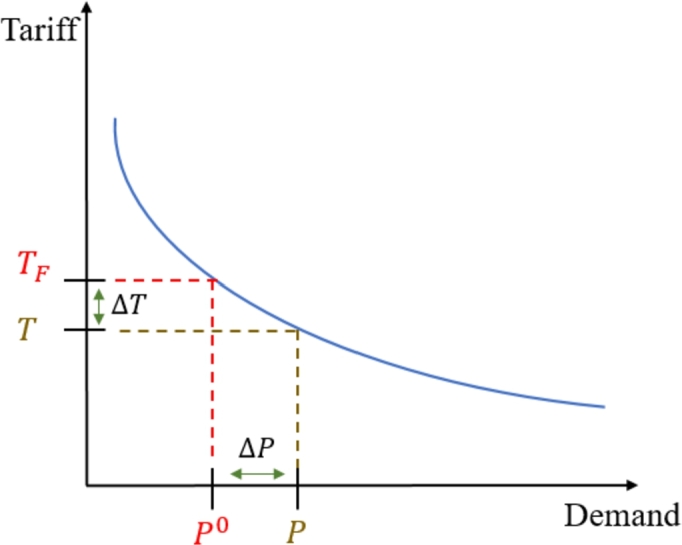
Load demand curve.

The consumer response is usually described through the price elasticity of demand. Price elasticity is a normalized measure used in economics to evaluate a commodity's demand changes according to the changes in its price [30]. Hence, price elasticity is a relative measure to facilitate the calculation of the consumers' response and is defined as demand sensitivity with respect to the price [31]. We will use price elasticity to model the consumer's response. The relationship between the demand and the price is defined by the demand curve [32], [33], illustrated in Fig. 5.

Demand price elasticity can be evaluated as $\xi =\frac{\Delta P}{\Delta T}\frac{T_{F}}{P^{0}}$, where $\Delta P=P-P^{0}$ and $\Delta T=T-T_{F}$ are demand change and price change, respectively; $P^{0}$ and $T_{F}$ indicate the initial electricity consumption and the flat electricity tariff before implementing the ToU electricity tariff, respectively. After the latter's implementation, the electricity consumption and the ToU tariff are *P* and *T*, respectively. The curve is linearized around the point ($P^{0},T_{F}$) as in Eq. (1)

(1) $$\overset{¯}{P}=P^{0}\left(1+\xi \frac{\Delta T}{T_{F}}\right),$$

where *ξ* is referred to as the elasticity coefficient.

ToU tariffs introduce multiple periods during the day with different prices; hence the demand may shift from one period to multiple other periods. Therefore we use self-elasticity and cross-elasticity coefficients to describe the relationship between energy demand and electricity price in different periods. It is assumed that the overall energy consumption during the day remains constant.

The self-elasticity coefficient $\xi_{ii}$ indicates the effect of price change of time period *i* on the demand of the same period. The cross-elasticity coefficient $\xi_{ij}$ shows how the demand changes in time period *i* when the price at time period *j* changes. For multi-period ToU tariffs, Eq. (1) becomes ${\overset{¯}{P}}_{i}=P_{i}^{0}+P_{i}^{0}\left(\sum_{j=1}^{C}\xi_{ij}\frac{T_{j}-T_{F}}{T_{F}}\right)$, where *C* is the number of daily periods of the ToU electricity tariff. Thus CBM can be arranged in a matrix form in Eq. (2)

(2) $$\overset{¯}{P}=\left(I+\Xi \frac{\Delta T}{T_{F}}\right)P^{0},$$

where $\overset{¯}{P}={\left[{\overset{¯}{P}}_{1},\ldots ,{\overset{¯}{P}}_{C}\right]}^{T}$ is the vector of electricity consumption by the household after implementing the ToU tariff with *C* tariff periods over the day. The price elasticity matrix $\Xi \left[\xi_{ij}\right]$ contains self-elasticity coefficients of all *C* periods in the diagonal and the cross-elasticity coefficients between the periods in the off-diagonal. The identity matrix *I* is of size C×C, $P^{0}={\left[P_{1}^{0},\ldots ,P_{C}^{0}\right]}^{T}$ is the initial electricity consumption, and $\Delta T=\text{diag}\{\Delta T_{1,F},\ldots ,\Delta T_{C,F}\}$ with $\Delta T_{j,F}=T_{j}-T_{F}$. Similar models are used for each cluster with a different number of periods. The total energy consumption remains unchanged as the tariff changes, hence $\sum_{j=1}^{C}{\overset{¯}{P}}_{j}=\sum_{j=1}^{C}P_{j}^{0}$. This also represents a constraint on the sensibility coefficients.

## 3 Genetic algorithm-based optimal design of ToU electricity tariffs

The main objective of the UC is to maximize its profits by optimizing the match between the procurement costs and the retail price of electricity. Thus, reducing electricity consumption during peak times is vital to achieving the objective above. An appropriate DSR program implemented on the consumer-side has been proposed before [4], [34] and demonstrated the control of the operations of the household's appliances (loads shifting, controlling the HVAC systems) to avoid running in on peaks reduces the power peaks. Moreover, the consumers are driven by their profits (monetary and comfort); thus, they react to a DSR program that guarantees financial incentives while maintaining their energy consumption preferences. The ToU electricity tariff design is formulated as a non-linear optimization problem which is solved here using genetic algorithms. Due to the high complexity of the optimization framework (many local optima) and the wide range of searches for the global optimal (alternating individuals thanks to the genetic operators), the GA is chosen to handle the multiple decision variables and numerous constraints effectively.

Without the loss of generality, the ToU tariff optimization is presented here for the three eligible consumer categories N=3 (high, medium-high, average) as discussed in Section 2. The result is the set of tariff periods and the prices for each category. It is also assumed that the UC may calculate the average consumption in one day of each cluster in the eligible categories and for each category based on the historical consumption data. These average values are referred to as baseline consumptions.

**Figure 6:**
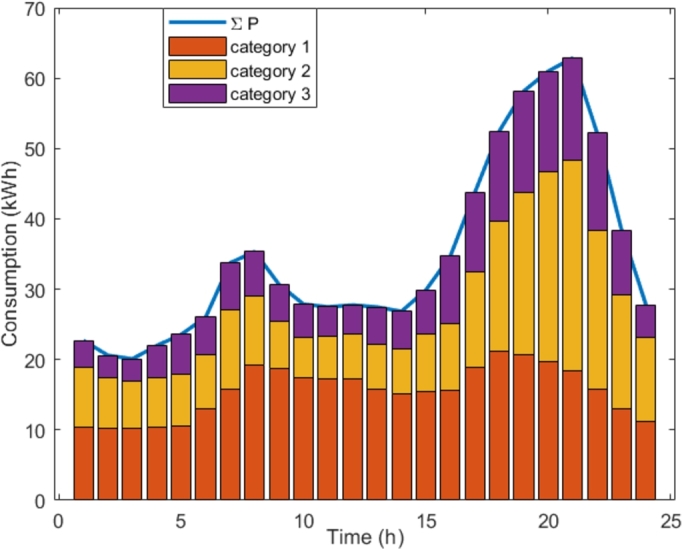
Average Monday consumption of all consumers in each eligible category and their contribution to each period (City A).

To justify our novel approach, namely a different ToU tariff for each consumer category, let us present a simple analysis of the contributions of the categories to power peaks. Fig. 6 illustrates the contribution of the categories eligible for the DSR program in City A, and each consumer category contributes differently to the overall on-peak period from 17 h to 22 h. The total energy consumed in this period by the three categories is 330.39 kWh. The categories 1, 2, and 3 contribute with 34.75%, 40.69% and 24.56%, respectively.

As different ToU tariffs are generated for each consumer category, some notations introduced in Subsection 2.2 are revisited. We specify the total consumption vector for the households of category *i* (i=1,…,N) after (resp. before) the application of ToU tariff as ${\overset{¯}{P}}^{i}={\left[{\overset{¯}{P}}_{1}^{i},\ldots ,{\overset{¯}{P}}_{C_{i}}^{i}\right]}^{T}$, (resp. $P^{0i}={\left[P_{1}^{0i},\ldots ,P_{C_{i}}^{0i}\right]}^{T}$), where $C_{i}$ is the number of tariff periods for category *i*.

### 3.1 Time-of-use tariff period selection

**Figure 7:**
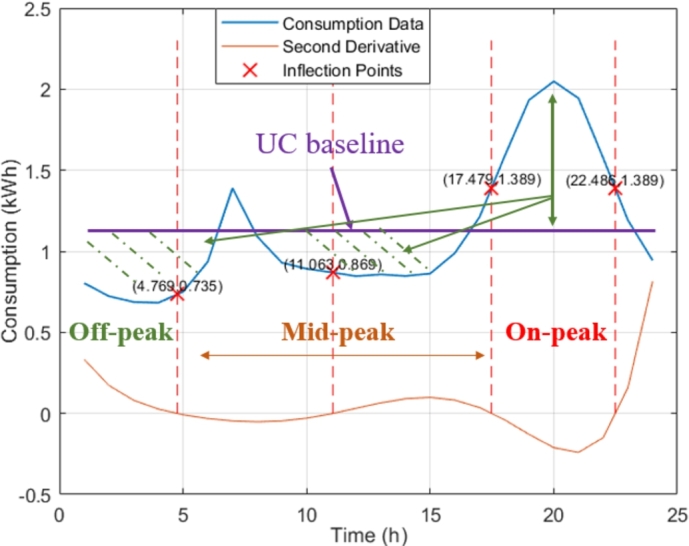
Labeled tariff periods (Category 3, City A).

Tariff periods can have different durations from one category to the other. We consider inflection points of the average demand curve of each category (rounded to the nearest integer hour) as candidates to separate the different tariff periods. To illustrate our approach, let us consider Fig. 7 showing the average consumption curve of category 3 of City A with the inflection points. The UC baseline represents the equivalent average energy consumption, and we denote by ${\underline{P}}_{j}^{i}$ the total baseline consumption of category *i* and tariff period *j*.

Next, each period is labeled from the set {Off-peak, Mid-peak, On-peak} using Table 2.

**Table 2:** Labeling Rules in Consumer Category *i* and for The Tariff Period *j* (*j* = 1,…,*C*_*i*_).

*Label*	*Rule*
*Off-peak*	$P_{j}^{0i}<(1-\lambda_{1}){\underline{P}}_{j}^{i}$
*Mid-peak*	$(1-\lambda_{1}){\underline{P}}_{j}^{i}<P_{j}^{0i}<(1+\lambda_{2}){\underline{P}}_{j}^{i}$
*On-peak*	$(1+\lambda_{2}){\underline{P}}_{j}^{i}<P_{j}^{0i}$

The parameters $\lambda_{1}$ and $\lambda_{2}$ ($\lambda_{1}\in (0,1)$, $\lambda_{2}>0$) determine the consumption range for the different labels. Their value can be different for each category. If two neighboring periods are labeled similarly, they are combined and considered a single tariff period so that the corresponding $C_{i}$ decreases. Recall that we consider the day's first and last tariff periods as neighboring periods. Taking the example presented in Fig. 7, three periods are finally labeled. The same algorithm is performed for the remaining eligible categories.

### 3.2 Time-of-use price optimization

Let $T_{tag}^{i}$ denote the tariff of the three different periods for category *i* (tag∈ {Off-peak, Mid-peak, On-peak}, i=1,…,N) so that $T_{\text{Off-peak}}^{i}<T_{\text{Mid-peak}}^{i}<T_{\text{On-peak}}^{i}$. We also need to reformulate Eq. (2) for the consumer category *i* (i∈1…N) as in Eq. (3)

(3) $${\overset{¯}{P}}^{i}=\left(I+\Xi^{i}\frac{\Delta T^{i}}{T_{F}}\right)P^{0i},$$

where $\Delta T^{i}=\mathrm{diag}\{T_{1}^{i}-T_{F},\ldots ,T_{C_{i}}^{i}-T_{F}\}$ so that $T_{j}^{i}\in \{T_{\text{Off-peak}}^{i},T_{\text{Mid-peak}}^{i},T_{\text{On-peak}}^{i}\}$.

In our approach, the UC seeks to minimize the difference between two cost components. The first component is the cost required to purchase energy on the market, denoted by Costs@UC. The second component is the income obtained applying the ToU tariff, denoted by Income@UC.

We consider first the costs of energy purchase. The early purchase of cheap energy is made at the price of $T_{F}^{UC}$ and uses a predicted consumption profile. Without loss of generality, we assume that the predicted consumption is constant and calculated using the average consumption ${\underline{P}}_{j}^{i}$. If the actual consumption is larger than the predicted one, extra energy has to be purchased at a unit cost of $T_{H}^{UC}$. Therefore, the energy purchase costs from the market are represented by Eq. (4)

(4) $$Costs@UC=\sum_{i=1}^{N}\sum_{j=1}^{C_{i}}\left(T_{F}^{UC}\left({\underline{P}}_{j}^{i}+{\underline{\Delta P}}_{j}^{i}\right)+T_{H}^{UC}{\bar{\Delta P}}_{j}^{i}\right),$$

such that ${\bar{\Delta P}}_{j}^{i}=\max({\overset{¯}{P}}_{j}^{i}-{\underline{P}}_{j}^{i},0)$ and ${\underline{\Delta P}}_{j}^{i}=\min({\overset{¯}{P}}_{j}^{i}-{\underline{P}}_{j}^{i},0)$.

Let us recall that the calculation of ${\overset{¯}{P}}_{j}^{i}$ involves the CBM reformulated as Eq. (3). The income component for the UC is given in Eq. (5)

(5) $$Income@UC=\sum_{i=1}^{N}\sum_{j=1}^{C_{i}}{\overset{¯}{P}}_{j}^{i}\cdot T_{j}^{i},$$

where $T_{j}^{i}\in \{T_{\text{Off-peak}}^{i},T_{\text{Mid-peak}}^{i},T_{\text{On-peak}}^{i}\}$.

The consumers' incentive to participate in the DSR program is the amount they can save by rescheduling a part of their consumption. The incentive component is denoted by Economy@Cons and represented in Eq. (6)

(6) $$Economy@Cons=\sum_{i=1}^{N}\sum_{j=1}^{C_{i}}\left(({\underline{P}}_{j}^{i}\cdot T_{F})-({\overset{¯}{P}}_{j}^{i}\cdot T_{j}^{i})\right).$$

Hence, the cost function (i.e., the objective function for the GA) reads in Eq. (7)

(7) $$F=Costs@UC-\alpha_{1}Income@UC-\alpha_{2}Economy@Cons,$$

where $\alpha_{1}$ and $\alpha_{2}$ are weights determining how the benefit of the reduced overconsumption is shared between the UC and the consumers. The weighting factor values are fixed and chosen to balance the shares, and their variation effect on the DSR program benefit is not addressed here.

As mentioned earlier, it is assumed that the total consumption of the households does not change due to the application of ToU tariffs. A further constraint is that all tariffs must be positive; however, unlike the existing methods, there is no need to limit the upper bound of the tariffs, hence the constraint is represented in Eq. (8)

(8) $$\sum_{j=1}^{C_{i}}{\overset{¯}{P}}_{j}^{i}=\sum_{j=1}^{C_{i}}P_{j}^{0i},\;T_{j}^{i}>0\;\;i=1,\ldots ,N.$$

The optimization problem is to minimize Eq. (7) subject to Eqs. (3) and (8).

## 4 Optimal time-of-use tariffs based on consumption data

Optimal ToU tariffs are determined for the categories identified in Section 2 so that the tariff periods are obtained according to Section 3. The prices are normalized to $T_{F}$ and chosen as $T_{F}^{UC}<T_{H}^{UC}<T_{F}$. GA-based optimization is performed, and Matlab's Global Optimization Toolbox (version 3.4.4) is used with standard options.

The price elasticity coefficients should be available to calculate the changes in load demand using the proposed CBM in Section 2.2. The elasticity coefficients can be calculated if a field measurement of the consumption after implementing the optimal ToU tariffs is conducted over a period of time. Since we have no data to determine the price elasticity coefficients, updated values of those used in [28] were selected to match the consumption in Hungary. Profitability robustness analysis and a simple identification method are presented in Section 5 for completeness.

**Figure 8:**
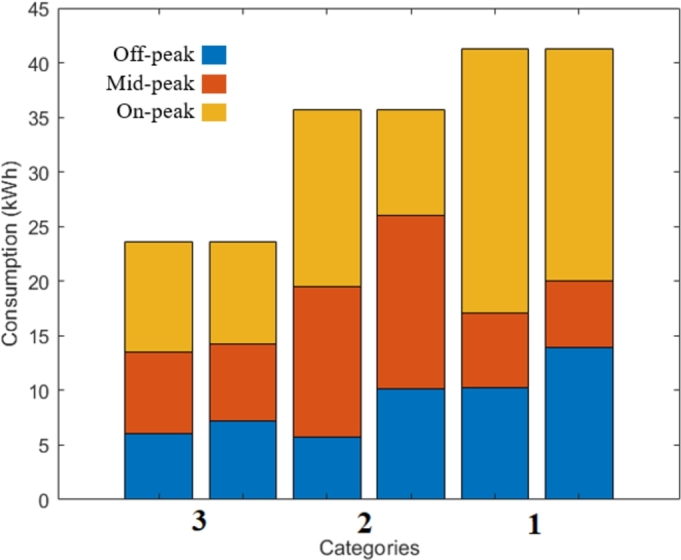
Total consumption of the eligible categories (average: 3, medium-high: 2, and high: 1) and the before and after implementing the ToU tariffs in City A.

Table 3 shows the optimal tariffs (City A, winter season). The resulting change in the distribution of the electricity consumption is presented in Fig. 8 for the consumer categories concerned. As it can be seen, three different ToU tariffs with different structures were obtained in City A. Thanks to the categorization phase, which in some existing literature has been skipped, only one ToU tariff structure is introduced for the same consumer population. Let us remark that the consumers in the 2^nd^ category get the highest tariff-based incentive to shift their consumption as it is the category consuming the most to the overall power peak. However, all the consumer categories benefit from a reduced electricity bill, which is different from the existing literature, where not all categories participating in the demand-side program can have reduced bills. In our strategy, we target the consumer category that contributes the most to the overall power peak, which is not performed in some earlier pricing strategies. Therefore, a significant amount (between 10−30%) of the on-peak load is shifted to off-peak and mid-peak periods.

**Table 3:** Tariffs in City A (Normalized to *T*_*F*_, Rounded to Two Decimals).

	*Off-peak*	*Mid-peak*	*On-peak*
*category* 1	0.30	1.08	1.45
*category* 2	0.16	0.52	2.43
*category* 3	0.59	1.12	1.23

**Figure 9:**
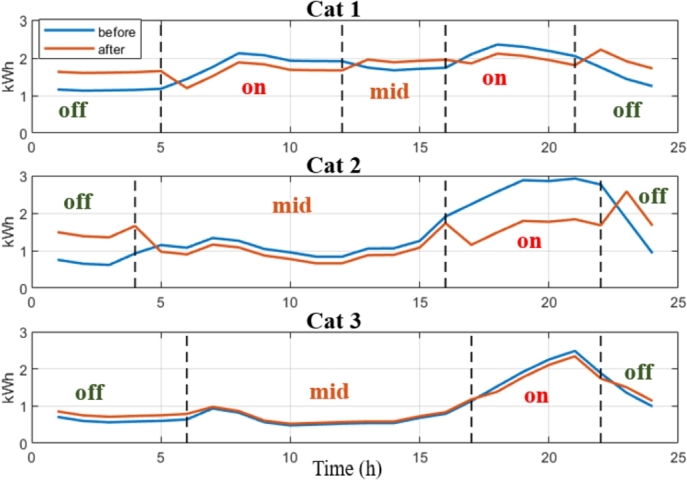
Average consumption curve for the three categories before and after implementing the ToU tariffs in City A.

It is assumed that the load shifted from the on-peak period is distributed almost uniformly in the other periods to prevent the appearance of new power peaks. For this purpose, an appropriate scheduling algorithm must be implemented on the consumer side by managing the operations of the shiftable appliances. These algorithms are not addressed here; however, such solutions were proposed in [35] recently and by the authors in [4]. Fig. 9 shows the change in the average hourly load curve using the ToU tariff.

Taking the average consumer in all categories, we can calculate the gains of the UC and the consumers using the optimal ToU tariffs. The results are shown in Table 4. Different from the presented literature, in our pricing strategy, the ToU periods are determined dynamically, resulting in better handling of each consumer category's behavior to reduce their bills. Moreover, the weighting factors introduced in the cost-based objective function guarantee a winning situation for all categories participating and the UC, where their values can show how the marginal gain is distributed among all parties, which was not addressed in the previous literature. The values in Table 4 show that the reduced overconsumption benefited the UC and the consumers of all categories. The overall power peaks are reduced by roughly 20%, which also benefits the energy distribution infrastructure.

**Table 4:** Average Profits Per Consumer Before and After Implementing The DSR Program (City A, Winter).

	*gain*	*%*	*shifted load %*
*UC*	1.15	2.54	19.77
*Category* 1	1.09	2.64	12.16
*Category* 2	1.81	5.08	32.17
*Category* 3	0.35	1.46	11.80

## 5 Profitability robustness and parameter identification

Let us recall that the price sensibility parameters of the matrix $\Xi^{i}$ in the CBM, Eq. (3), are supposed to be known to find the optimal ToU tariff for each consumer category. If the real price sensibility is different, then the ToU tariff may not be optimal. Hence it is useful to analyze the profitability robustness of the tariff against the uncertainty of the elements of $\Xi^{i}$.

For the sake of simplicity, we assume two price periods and one consumer category so that the nominal values are denoted by $\Xi_{0}=\left[\xi_{ij,0}\right]$, (i,j∈{1,2}). Let us assume the diagonal elements of $\Xi_{0}$ are $\xi_{11,0}=-0.7$ and $\xi_{22,0}=-0.5$ and remark that the other elements can be obtained by the consumption conservation assumption in Eq. (8).

**Figure 10:**
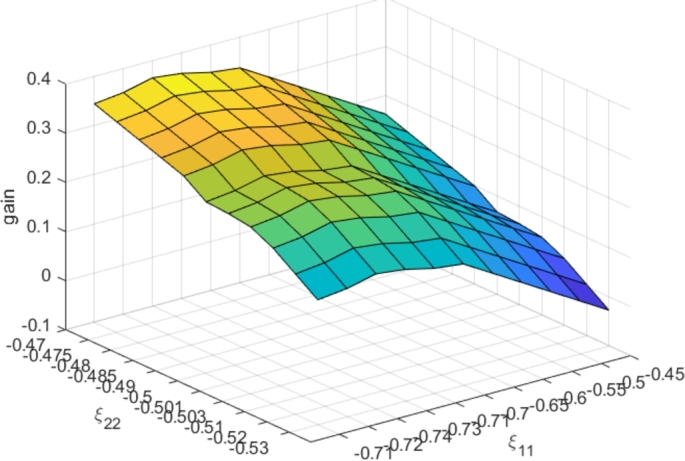
Dependence of UC gains on price sensibility parameter variations.

**Figure 11:**
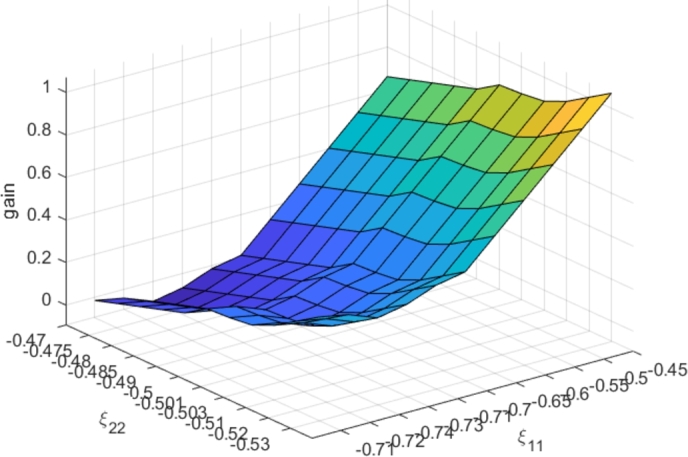
Dependence of consumer gains on price sensibility parameter variations.

The optimal ToU tariffs are calculated for $\Xi_{0}$. To check the robustness of ToU profitability for both the UC and the consumers, we apply this calculated tariff using the CBM (Eq. (3)) with parameter values Ξ, different from $\Xi_{0}$, considering an uncertainty interval for each parameter: $\xi_{ij}\in [\xi_{ij,0}-{\underline{\xi }}_{ij},\xi_{ij,0}+{\overset{¯}{\xi }}_{ij}]$. In our example: ${\underline{\xi }}_{11}=0.04$, ${\overset{¯}{\xi }}_{11}=0.25$, ${\underline{\xi }}_{22}=0.03$ and ${\overset{¯}{\xi }}_{22}=0.03$. Considering category 3 of City A, the resulting UC and consumer gains are shown in Fig. 10 and Fig. 11, respectively. One may observe that the profitability remains robust (positive gains) even if the tariff is no longer optimal. Furthermore, a consumer might fall into a category that is not suitable for him (due to usage of the consumption data only in the categorization phase). The optimal ToU tariff assigned to his category will also efficiently save on his electricity bill. This is thanks to the wide positive gain range, even with uncertain consumer model behavior parameters.

Thanks to its smart metering infrastructure, the UC may use real consumption measurements over a limited time period to identify the price sensibility parameters after applying the ToU tariff. The identification of the elements of $\Xi^{i}$ is simply based on Eq. (3). Since the consumption conservation may not hold exactly, normalization of the energy consumption values (${\overset{¯}{P}}_{i}$ and $P^{0i}$) is necessary. This approach respects the consumers' privacy since no other data than the hourly consumption is needed, which is recorded automatically. Most of the existing literature identifies the consumer behavior model after collecting different consumption-related information (e.g., which appliances are on at which time, how many appliances are in the household, etc.). The consumer might see this unsolicited provision as a breach of his private data. The smart meter records the consumption with a usual granularity of 15 min. The information in this short time interval is unprecise to determine which appliances are operating. Moreover, the consumer will be free to react to the price signal as he pleases without unsolicited interventions.

To illustrate the identification process, let us reconsider the earlier example of this Section (category 3, City A with the nominal parameter values $\xi_{11,0}$, $\xi_{22,0}$). The optimal ToU electricity tariff prices are $T_{1}=0.8174$ and $T_{2}=1.5$. The initial demand load in each period is 17.50 and 9.83. Let us suppose that the newly measured consumption after introducing the ToU tariff is 19.78 and 6.80 for the two periods, respectively. The identified price elasticity values are $\xi_{11}=-0.72$ and $\xi_{22}=-0.49$, close to the initially used ones.

## 6 Conclusion

This paper presented a selective and optimal ToU pricing strategy. The strategy comprises the categorization of consumers so that different ToU electricity tariffs can be proposed for each category. The ToU tariff optimization uses a simple, price elasticity-based CBM. The suggested tariff scheme can be easily coupled with consumer-side scheduling algorithms. The effectiveness of the proposed model has been verified using real consumption data.

The optimal ToU tariffs succeeded in shifting 20% from the overall power peak by targeting the consumer category contributing the most to it. Hence, the UC has reduced its costs where its profit increased by 2.45% in one day, and one consumer can save up to 5% on his daily bill. The profits' robustness is guaranteed with parameter uncertainties in the consumer behavior model, which is essential in the ToU tariff design. The advantage of the presented approach is that the consumer's privacy is not violated since no additional information, other than the consumption, is needed to analyze and categorize the consumers and identify the consumer behavior model's parameters. Furthermore, the heuristic methods to categorize consumers and flexibly determine the ToU periods using each category's behavior are crucial to charge the consumers properly.

As a future research activity, the optimization framework can be easily extended to more complex consumption prediction schemes, ToU tariff structures, and improved consumer clustering and categorization techniques based on more complex features. Since the ToU tariff design depends on the consumer behavior model parameters, an analysis of the time span they will remain unchanged will be made. An analysis of how the weighting factors in the cost function can affect the distribution of the gain margin on the consumers and the UC will be presented in future publications.

## Declarations

### Author contribution statement

Salma Taik: Conceived and designed the experiments; Performed the experiments; Analyzed and interpreted the data; Contributed reagents, materials, analysis tools or data; Wrote the paper.

Bálint Kiss, Dr: Contributed reagents, materials, analysis tools or data; Wrote the paper.

### Funding statement

This work was supported by the Ministry of Innovation and Technology of Hungary from the 10.13039/501100012550National Research, Development and Innovation Fund, financed under the TKP2021 funding scheme [BME-NVA-02].

### Data availability statement

Data included in article/supp. material/referenced in article.

### Declaration of interests statement

The authors declare no conflict of interest.

### Additional information

No additional information is available for this paper.
